# Cost-Effectiveness of Routine X-Rays After Central Venous Catheter Removal: A Value-Based Analysis of Post-Removal Complications

**DOI:** 10.3390/jcm14041397

**Published:** 2025-02-19

**Authors:** Martin Breitwieser, Teresa Wiesner, Vanessa Moore, Florian Wichlas, Christian Deininger

**Affiliations:** Department for Orthopedic Surgery and Traumatology, Paracelsus Medical University, 5020 Salzburg, Austriav.moore@salk.at (V.M.); f.wichlas@salk.at (F.W.); c.deininger@salk.at (C.D.)

**Keywords:** CVC, central venous catheter, CEA, cost-effectiveness analysis, removal, cost-per-outcome analysis, X-ray

## Abstract

**Background**: Healthcare systems worldwide are increasingly burdened by rising costs, growing patient demand, and limited resources. In this context, cost-effectiveness analysis (CEA) plays a vital role in evaluating the clinical value of medical interventions relative to their costs. Despite the lack of evidence supporting their necessity, routine post-removal chest X-rays for central venous catheters (CVCs) are still performed in some hospitals due to persistent misconceptions about their benefits. This study seeks to address these misconceptions by examining the costs of routine imaging through a cost analysis of complication detection rates in a large inpatient cohort, with the aim of highlighting the inefficiencies of this practice and promoting evidence-based approaches. **Methods**: A retrospective cohort analysis was performed across four university hospitals in Salzburg, Austria, including 984 CVC removals conducted between 2012 and 2021. Comparisons were made between X-rays after primary catheter insertion and post-removal X-rays to isolate complications specifically associated with CVC removal. A simple cost-per-outcome analysis, a subtype of CEA, was chosen to determine the cost per complication detected. The approach incorporated activity-based costing, adjusted to 2024 price levels via the Austrian Consumer Price Index (CPI), to capture real-world resource utilization. **Results**: Complications related to CVC removal were identified in five cases (0.5%), including one catheter rupture due to self-removal, two failed removals, one hemothorax, and one case of intrathoracic bleeding. Of these, three complications were detected on X-rays, including a retained catheter fragment, signs of intrathoracic bleeding, and a hemothorax. Additionally, one asymptomatic patient had a likely incidental finding of a small pneumothorax, which required no intervention. The cost of routine X-rays was calculated at EUR 38.20 per X-ray, resulting in a total expenditure of EUR 37,588.80 for 984 X-rays. This corresponds to EUR 7517.76 per detected complication (*n* = 4). The odds of detecting a complication on an X-ray were 193 times higher in symptomatic patients than in asymptomatic patients (*p* < 0.001). **Conclusions**: This study confirms that complications following CVC removal are rare with only five detected cases. Routine imaging did not improve clinical decision-making, as complications were significantly more likely to be identified in symptomatic patients through clinical evaluation alone. Given the high financial cost (EUR 37,588.80 for 984 X-rays, EUR 7517.76 per detected complication), routine post-removal X-rays are unnecessary in asymptomatic patients and should be reserved for symptomatic cases based on clinical judgment. Adopting a symptom-based imaging approach would reduce unnecessary healthcare costs, minimize patient radiation exposure, and optimize resource allocation in high-volume procedures such as CVC removal.

## 1. Introduction

Healthcare systems worldwide face significant challenges that strain resources and threaten the sustainability of quality patient care. An aging global population has increased the prevalence of chronic diseases and the demand for medical services, while advances in medical technology, though offering new treatment possibilities, come with substantial costs. The aftermath of the COVID-19 pandemic has further exposed vulnerabilities in resource allocation and emergency preparedness, underscoring the need for optimization [1]. These pressures make it essential for healthcare systems to prioritize interventions that are not only clinically effective but also economically sustainable, following the principles of value-based medicine [2]. Cost efficiency has become a key consideration in healthcare decision-making, ensuring that limited resources are used to maximize patient outcomes without unnecessary expenses [3].

Over the last two decades, cost-effectiveness analysis (CEA) has become a vital tool for identifying healthcare interventions that provide the best value by balancing costs and clinical outcomes. Its application to routine medical procedures can help minimize unnecessary expenditures while ensuring patient safety and optimizing resource allocation [4,5,6,7]. Routine imaging, often performed without clear clinical indications, exemplifies practices that can lead to unnecessary expenditures without consistently enhancing patient management or diagnostic accuracy. This underscores the importance of targeted approaches to optimize resource use and reduce wasteful practices [8,9,10].

Simple cost-per-outcome analysis, a subtype of CEA, can be utilized to evaluate the cost of detecting a single complication in routine imaging. By calculating the cost per complication detected, this approach can directly assess its financial efficiency in a high-volume setting. One of the key advantages of this cost-per-outcome method is its clarity and direct applicability, providing clinicians and policymakers immediate insight into whether the number of complications identified justifies the cost of routine imaging.

A key example of routine imaging in some hospitals is the chest X-ray performed after central venous catheter (CVC) procedures. Complications following CVC removal are rare and can be categorized as either mechanical or thrombotic events. Mechanical complications, such as air embolism and catheter fracture, occur infrequently and represent the most immediate concerns [11,12,13,14]. Thrombotic complications, including embolic events caused by dislodged thrombi, have been documented but remain uncommon after CVC removal [15,16].

Among these potential complications, the detection of a fractured catheter is the only issue that can reliably be identified through a chest X-ray. Proper documentation of catheter length during insertion and careful verification upon removal significantly reduce the risk of undetected fractures. Accordingly, chest X-rays can be considered if a discrepancy in catheter length is observed [17]. Pneumothorax, although often incorrectly feared by clinicians, is not directly associated with CVC removal. The literature contains only one reported case, which was not conclusively linked to the removal procedure [18].

Despite consistent findings in the literature, some hospitals continue to perform routine chest X-rays after CVC removal due to persistent misconceptions about their benefits, aiming to prevent severe outcomes and the potentially higher costs associated with delayed detection. However, such proactive imaging is unlikely to be justified, as overdiagnosis and overtreatment contribute to both financial and societal burdens without consistently improving patient outcomes [8,19]. This overuse of imaging is part of a broader issue, with up to one-third of healthcare spending wasted on duplicative or unnecessary procedures, often driven by defensive medicine rather than evidence-based clinical indications [9,20]. Unnecessary imaging often yields incidental findings that require further follow-up, adding to healthcare expenditures without demonstrable benefits [8]. Moreover, overdiagnosis can subject patients to treatments for conditions that do not warrant intervention, thus increasing the risk of harm [21].

The current literature provides no evidence to support the necessity of routine chest X-rays for asymptomatic patients following CVC removal. This study aims to underscore these findings by highlighting the inefficiency and financial burden associated with routine imaging after CVC removal while advocating for the adoption of evidence-based guidelines to optimize clinical practices.

## 2. Materials and Methods

This retrospective cohort study was conducted at the Salzburger Landeskliniken, a network of four university hospitals in Salzburg, Austria. The hospital network includes the Universitätsklinikum Salzburg, the largest hospital in the region with 1500 beds; the Christian-Doppler-Klinik, a specialized neurological and neurosurgical center with approximately 200 beds; the Krankenhaus Hallein, a regional hospital with 200 beds; and the Krankenhaus Tamsweg, a smaller community hospital with 100 beds. The overall ICU bed capacity across the network is approximately 150 beds. These hospitals serve a mixed population that includes both high-acuity and general-care patients.

The study analyzed chest X-rays performed between 1 January 2012 and 31 December 2021, following CVC removals from the internal jugular and subclavian veins. The primary outcome measure was the cost-efficiency of routine imaging following CVC removal, evaluated separately for symptomatic and asymptomatic patients. This was assessed by comparing the total cost of X-rays with the clinical benefit, quantified as the number of complications detected. Secondary outcomes included (1) the identification and classification of complications and (2) the analysis of associated risk factors contributing to these complications.

### 2.1. Data Gathering

The initial dataset was retrieved from electronic health records (EHR) using specific keywords related to CVC procedures, consisting of 26,109 records. Each record included patient demographic data (age, gender), the date of the X-ray, the department of admission, the radiologist’s report, and the clinician’s request for the X-ray. All sensitive patient information was then de-identified to ensure compliance with institutional ethical guidelines, as approved by the Salzburger Ethics Committee. The responsibility for CVC removal in these hospitals lies primarily with treating physicians or trained nursing staff, guided by general best practices. Routine chest X-rays after CVC removal are not universally mandated across all settings, contributing to variability in the number of X-rays identified in the EHR.

The inclusion criteria comprise the following:

- Chest X-rays performed within 12 h of CVC removal;
- Previous chest X-ray after CVC insertion for comparison;
- Patients aged 18 years or older at time of CVC removal;
- The exclusion criteria comprise the following:
- Incomplete or missing data;
- Primarily misplaced CVCs during insertion;
- Placement or removal of other drains on the same side of the body as the CVC removal;
- Patients under 18 years of age.

After applying these criteria and a rigorous screening process by three physicians to identify X-rays after CVC removal, 984 X-rays of 926 different adult patients were included in the final analysis, and further data on complications and symptoms after CVC removal were gathered from EHR. One patient was excluded from the analysis due to an initially misdiagnosed pneumothorax on the post-removal chest X-ray. However, this diagnosis was ruled out later the same day following a chest CT scan, which confirmed the absence of a pneumothorax.

### 2.2. Calculation of Cost-Effectiveness

A simple cost-per-outcome analysis was chosen to evaluate the financial and clinical effectiveness of routine chest X-rays following CVC removal. This analysis aims to determine the cost-effectiveness of these X-rays by calculating two primary metrics: the number needed to screen (NNS) to detect a single complication and the cost per complication detected. These calculations provide a straightforward measure of the balance between healthcare expenditures and clinical benefits, highlighting whether routine imaging offers sufficient value to justify its use.

By integrating activity-based costing and inflation-adjusted price levels for 2024, this analysis ensures that the financial costs reflect real-world resource utilization. This includes personnel, direct material, and overhead costs.

To determine the cost of a single chest X-ray, the current Austrian performance-based hospital financing values for 2024 from the “Leistungsorientierte Krankenanstaltenfinanzierung” (LKF) were first identified to establish the relevant lump sum payment. Building on these values, an activity-based costing approach was then applied to more accurately reflect the resources consumed. The process began by recording the amount of time each professional group (e.g., radiology technicians, radiologists) devoted to a single X-ray, thereby enabling personnel costs to be allocated proportionally based on time. Material costs, including both direct consumables (e.g., contrast agents) and overhead materials (e.g., general maintenance and shared supplies), were assigned accordingly. Direct materials used during each examination were attributed in full to that particular X-ray, while overhead material expenses were evenly distributed across all examinations.

This cost calculation can be expressed using the following formula:

(1) $$Cost_{Xray}=\left(\underset{j}{\sum }T_{j}\times C_{j}\right)+M_{direct}+\frac{M_{overhead}}{N}$$

Here, *j* represents each professional group involved in providing an X-ray, *T_j_* represents the time in minutes required by this professional group j per chest X-ray, and *C_j_* represents the corresponding cost per minute (including wages and benefits). *M_direct_* captures the total direct material costs per X-ray (e.g., single-use items and contrast agents), while *M_overhead_* refers to overhead material costs distributed evenly among all X-rays performed in this time (N). By employing this formula, the resulting figure provides a transparent and accurate estimate of the cost per chest X-ray, ensuring that both personnel and material expenses are properly accounted for.

To ensure that the calculated costs accurately reflected present-day economic conditions, the estimated expenses derived through activity-based costing were further adjusted to 2024 price levels using the Austrian Consumer Price Index (CPI).

(2) $$Cost_{Xray\_2024}=Cost_{Xray}\times CPI_{factor}$$

This adjustment aligns the figures from the 2012–2022 timeframe with current monetary values, mitigating the impact of inflation and other price fluctuations. By applying the CPI as a scaling factor, the standardized cost of EUR 38.20 per chest X-ray—incorporating both routine examinations and those conducted due to complications—accurately reflects the 2024 economic landscape, thereby providing a more robust foundation for the subsequent cost-effectiveness analysis.

The total cost of post-removal X-rays was then calculated using the following formula:

(3) $$Total\_Cost=N\times Cost_{Xray\_2024}$$

In this calculation, *Total_Cost* represents the total cost of post-removal X-rays, *N* is the total number of chest X-rays performed, and *Cost_Xray_2024_* is the cost of a single chest X-ray in 2024.

The *NNS* was calculated to estimate how many routine post-removal chest X-rays were required to detect one complication related to CVC removal. The formula used for this calculation was

(4) $$NNS=\frac{N}{N_{complications}\;}$$

In this formula, *NNS* is the number of X-rays required to detect one complication, *N* is the total number of chest X-rays performed, and *N_complications_* is the number of X-rays that showed complications related to CVC removal.

From the dataset, the number of X-rays that revealed complications related to CVC removal (such as pneumothorax, excessive bleeding, or mechanical complications) was identified. This served as the primary outcome measure in assessing the clinical value of post-removal X-rays. The cost-effectiveness of routine X-rays after CVC removal was determined using the following calculation:

(5) $$Cost\;per\;Complication\;Detected=\frac{Total\_Cost}{N_{complications}}\;$$

In this calculation, *Total_Cost* represents the total cost of post-removal X-rays, and *N_complications_* is the number of X-rays that showed complications related to CVC removal. This calculation estimates the financial cost required to detect one post-removal complication through routine imaging.

### 2.3. Logistic Regression Analysis

To assess potential risk factors associated with complications following central venous catheter (CVC) removal, a multivariable logistic regression analysis was performed. The selected predictor variables included sex, age, CVC dwell time, catheter placement site (Jugularis or Subclavia), and catheter placement side (right or left). These variables were chosen based on their established or plausible clinical relevance, as supported by the prior literature and clinical reasoning. To mitigate overfitting, the number of predictors was limited to five based on clinical relevance, interaction terms were excluded to maintain model simplicity and model performance was assessed using residual deviance and AIC. Additionally, all predictors were included in the multivariable model, and their independent contributions were validated through univariable regression analyses to ensure robustness and prevent spurious associations.

### 2.4. Statistical Methods

All statistical analyses were conducted using RStudio (version 2024.04.2) and Microsoft Excel 2016.

To assess the effectiveness of routine post-removal X-rays for detecting complications after CVC removal, a Fisher’s Exact Test was employed to evaluate the association between complications and X-ray findings in both symptomatic and asymptomatic patients. This test allowed us to assess whether there was a significant association between complication occurrence and X-ray findings in both patient groups.

### 2.5. Ethical Considerations

Ethical approval for this study was granted by the Salzburger Ethics Committee, ensuring compliance with ethical standards and patient confidentiality on 30 March 2022 (ethical approval code 1032/2022). All participants’ data were anonymized, and no new primary data were collected for this analysis.

## 3. Results

### 3.1. Patient Demographics

This study included 984 X-rays of 926 patients, with ages ranging from 18 to 101 years. The median age was 72 years, and the mean age was 68.5 years, indicating a predominantly older patient population. The gender distribution was relatively balanced, with 503 female patients (54.3%) and 423 male patients (45.7%).

Routine chest X-rays after CVC removal were performed across 34 departments spanning various specialties, illustrating the broad clinical reach of routine post-procedural X-rays. The departments included intensive care medicine, internal medicine, trauma surgery and orthopedics, neurology, pulmonology, geriatrics, and cardiology, among others. The highest number of X-rays came from the Department of Internal Medicine I (436 X-rays, 44.3%) and the Department of Trauma Surgery and Orthopedics (242 X-rays, 24.6%).

Of the 926 patients included in the study, the majority of 871 patients (94.1%) had a single CVC removed during their hospital stay. However, 52 patients (5.6%) required two CVC removals, and 3 patients (0.3%) had three consecutively inserted CVCs removed during prolonged hospital admissions, with standard X-rays performed after each removal to check for complications.

CVC placement sites in this study included 718 in the internal jugular vein (73.0%) and 266 in the subclavian vein (27.0%). Moreover, 707 CVCs were positioned on the right side (71.9%), while 277 were placed on the left (28.1%). The median CVC dwell time was 9 days, with a mean of 11.35 days and a range of 0 to 63 days, where a dwell time of 0 days indicates that the CVC was removed on the same day it was inserted.

### 3.2. CVC Removal and Complications

The most common reason for CVC removal was the planned discontinuation of therapy or patient discharge, accounting for 730 cases (70.7%). Catheter side-changes were necessary in 147 cases (14.2%), while 60 patients (5.8%) either removed their CVCs themselves or experienced accidental dislodgement. In 47 cases (4.6%), catheter removal was prompted by suspected or confirmed local infections or thrombosis.

Overall, 966 CVC removals (98.2%) were uneventful, with patients showing no discomfort or symptoms post-procedure, highlighting the general safety of the procedure. Complications and adverse events related to the removal of the CVC were reported in 18 cases (1.8%) on the physician’s X-ray requests. Among these, bleeding occurred in 4 cases (0.4%), typically resulting from vessel injury during removal or due to anticoagulation therapy. Mechanical complications were noted in 3 cases (0.3%)—2 instances where the catheter could not be removed without surgical intervention and 1 case where a catheter fragment remained in the vein because the patient forcibly removed the catheter. Respiratory issues, the most frequent occurrence following CVC removal, were stated in 11 cases (1.0%) on the X-ray requests and involved breathing difficulties, coughing, or drops in oxygen saturation, which required further medical intervention (see Table 1).

Among the 18 symptomatic cases, three complications were identified on post-removal X-rays. One patient had a retained catheter fragment approximately 15 cm in length, while another showed signs of intrathoracic bleeding. The third patient developed a hemothorax following CVC removal, attributed to an acute arterial bleed from the subclavian artery, necessitating immediate medical intervention. For the other 15 symptomatic patients, no radiological findings for the adverse event reported on the X-ray request could be found.

Among the asymptomatic patients (*n* = 966), only one case of a small pneumothorax was incidentally detected on a routine post-removal X-ray. This finding was minor, required no medical intervention, and had no confirmed causal relationship with CVC removal.

### 3.3. Cost-Efficiency Analysis

The *NNS* showed that approximately 246 routine post-removal chest X-rays are required to detect a single complication associated with CVC removal in the overall cohort. When considering asymptomatic patients specifically, this figure rises to 966, reflecting the fact that among nearly one thousand asymptomatic individuals, only a single, most likely incidental finding was identified by routine imaging.

The total cost for the 984 post-removal X-rays was EUR 37,588.80. When assessing cost-effectiveness based solely on asymptomatic patients, only one questionable complication was identified in 966 cases, resulting in an effective expenditure of EUR 36,901.20 per detected complication. Furthermore, in this case, the finding required no intervention, and its direct relation to CVC removal could not be confirmed. Including the X-rays performed in symptomatic patients, a total of four complications were detected, corresponding to approximately EUR 9397.20 per complication. These findings indicate that the financial costs associated with routine post-removal X-rays are substantial relative to the low number of complications identified.

### 3.4. Analysis of Complication Detection

To assess the necessity of routine post-removal X-rays for detecting complications in symptomatic and asymptomatic patients, Fisher’s Exact Tests were performed on the dataset.

1. Odds Ratio: 193.0
2. 95% Confidence Interval: [18.97; 1963.83].

Among asymptomatic patients (*n* = 966), only one complication was found, which was minor and required no treatment. In symptomatic patients (*n* = 18), three complications were identified on X-rays. However, the majority of adverse events in this group were diagnosed based on clinical symptoms rather than imaging (see Table 2).

The odds of detecting a complication on an X-ray were 193 times higher in symptomatic patients compared to asymptomatic patients, which shows that complications were significantly more likely to be detected on X-rays in symptomatic patients compared to asymptomatic patients (*p* < 0.001). This confirms that symptoms are a strong indicator of complications, making routine imaging unnecessary for patients without symptoms.

## 4. Discussion

This study demonstrates that routine post-removal chest X-rays following CVC removal are associated with substantial financial costs relative to the low incidence of detected complications. This study utilized a simple cost-per-outcome method, a subtype of CEA, to evaluate whether the costs associated with routine post-removal chest X-rays are justified by the complications detected. By focusing on the cost per complication identified rather than broader metrics such as life-years gained or quality of life, this approach provided a straightforward assessment of tangible clinical outcomes. It also facilitated transparent comparisons with other imaging practices.

The standardized cost per X-ray, calculated at EUR 38.20 using an activity-based costing model adjusted to 2024 price levels through the Austrian CPI, compares favorably with prior studies that reported higher per-X-ray costs ranging from USD 51.80 to USD 183.00 [7,22,23]. However, the cost of EUR 9397.20 per detected complication underscores the economic inefficiency of this practice, particularly in asymptomatic patients, where the cost rises sharply to EUR 36,901.20 per detected complication. These findings align with prior studies indicating that routine imaging without clear clinical indications contributes significantly to unnecessary healthcare expenditures and does not consistently improve patient outcomes [8,19,24,25,26].

There is no universally accepted threshold for “cost per complication detected” or “cost per complication avoided” akin to the commonly referenced thresholds for cost-effectiveness based on cost per QALY [27]. Reviews of global health costs conclude that methodological heterogeneity and lack of transparency make it impossible to compare studies over setting and time, and several papers point to the need to develop standardized methods for cost estimation in global health [28].

Beyond financial considerations, routine imaging also increases patient exposure to ionizing radiation. Although a single chest X-ray delivers only 0.1 mSv of radiation, this exposure contributes to an annual medical radiation dose of approximately 0.4 mSv per individual [29,30]. Given the low diagnostic yield of routine imaging, particularly in asymptomatic patients, this additional radiation exposure appears unjustified.

Furthermore, healthcare workers are also subject to occupational radiation exposure, with radiologic technologists and physicians being among the most exposed, particularly in nuclear medicine and interventional radiology settings. While exposure levels have generally declined over the past decade due to improved safety measures, unnecessary imaging still contributes to cumulative radiation doses for medical staff, underscoring the importance of minimizing nonessential procedures [31].

In addition to radiation risks, routine chest X-rays have an environmental impact due to energy consumption and waste generation. A recent life cycle assessment found that each chest X-ray emits approximately 0.8 kg CO_2_, primarily from electricity use and the laundering of reusable materials such as sheets and pillowcases. Although this is lower than other imaging modalities like CT or MRI, the cumulative effect of unnecessary X-rays contributes to healthcare’s overall carbon footprint [32]. Moreover, scanners spend a significant amount of time in standby mode, consuming energy even when not in use. Optimizing scanner utilization and reducing unnecessary imaging would not only lower radiation exposure and costs but also decrease the environmental burden of healthcare operations. These findings emphasize the importance of symptom-driven imaging, which could reduce costs and radiation exposure while maintaining patient safety [33].

From a clinical perspective, this study supports the adoption of imaging protocols based on clinical judgment. Symptomatic patients typically present with signs such as respiratory distress or excessive bleeding, which provide sufficient diagnostic clues for appropriate management. In this study, clinical evaluation alone was adequate to identify complications, including excessive bleeding, mechanical issues (e.g., retained catheter fragments), and respiratory distress. In these cases, imaging merely confirmed what was already evident from the clinical presentation, adding no new information to guide management. As a result, routine X-rays in symptomatic patients rarely provide additional diagnostic value. This observation aligns with existing guidelines that question the necessity of routine imaging following uncomplicated CVC removals [34]. Furthermore, our statistical analysis demonstrated that clinical signs and symptoms are more reliable indicators of complications than X-ray findings in symptomatic patients.

In asymptomatic patients, routine X-rays yielded only one incidentally detected pneumothorax, which required no intervention and had no established causal relationship with CVC removal. This finding highlights the exceedingly low diagnostic yield of routine imaging in this subgroup, further questioning its justification.

Proper CVC removal techniques are essential for minimizing complications and reducing the need for imaging. The risk of an air embolism can be lowered by removing the catheter with the patient in a supine position and promptly sealing the skin breach with cyanoacrylate glue after local compression. Similarly, the risk of bleeding is best managed through rapid skin closure with cyanoacrylate glue rather than other empirical methods. Additionally, pre-removal ultrasound is recommended for high-risk patients to detect asymptomatic venous thrombosis before catheter removal [35]. Ultrasonography also provides a safer and more cost-effective alternative to routine chest X-rays, reducing both costs and radiation exposure [36,37]. It has demonstrated high accuracy in detecting complications such as pneumothorax after CVC insertion and is increasingly advocated as the preferred imaging method following CVC procedures [38,39,40]. Implementing these best practices can further minimize the need for post-removal imaging, reinforcing a symptom-based approach to patient management.

Building on these findings, the cost-efficiency analysis raises further questions about the economic value of routine post-removal X-rays. Given their high financial cost and low diagnostic yield, adopting a targeted, symptom-based approach would improve both economic and clinical efficiency, contributing to more value-based healthcare practices.

This study has notable strengths, including a large sample size, a long timeframe, and a rigorous cost-analysis methodology adjusted to current price levels. However, limitations include its retrospective design and reliance on data from a single center, which may reduce generalizability to other healthcare settings. Missing or incomplete data from electronic health records could introduce bias. Furthermore, the study focused solely on complications detectable by X-ray, excluding others such as infections, and did not directly compare alternative modalities like ultrasound.

## 5. Conclusions

This study confirms that complications following CVC removal are rare, with only five detected cases. Routine post-removal chest X-rays did not improve clinical decision-making, as clinical evaluation alone was sufficient for identifying complications in symptomatic patients.

Only three complications were detected on X-rays (a retained catheter fragment, signs of intrathoracic bleeding, and a hemothorax), while one likely incidental pneumothorax was found in an asymptomatic patient, requiring no intervention.

From an economic perspective, routine post-removal X-rays were associated with high costs and low diagnostic value. The total cost of 984 X-rays amounted to EUR 37,588.80, translating to EUR 7517.76 per detected complication. Given that routine imaging provided minimal additional clinical benefit, its widespread use represents an inefficient allocation of healthcare resources.

These findings suggest that post-removal imaging protocols should prioritize clinical judgment over routine application. Adopting a more targeted, symptom-based approach could significantly reduce unnecessary expenditures, minimize patient radiation exposure, and maintain high-quality care.

Future research should explore alternative imaging modalities, such as ultrasound, and validate these results through prospective, multi-center studies to ensure applicability across diverse healthcare and cost settings. By aligning imaging protocols with the principles of value-based medicine, healthcare systems can better allocate resources while safeguarding patient safety and sustainability.

## Figures and Tables

**Table 1:** Catheter-removal-related adverse event.

Adverse Events and Complications	Number of Cases	% of All Removals	% of All Complications
Bleeding	4	0.4%	22.2%
Mechanical complications	3	0.3%	16.7%
Respiratory issues	11	1.0%	61.1%

**Table 2:** Contingency table of post-removal complication detection on X-rays in symptomatic and asymptomatic patients.

	Symptomatic Patients	Asymptomatic Patients
Complication Detected on X-ray	3	1
No Complication Detected on X-ray	15	965

## Data Availability

The data presented in this study are available upon request from the corresponding author due to ethical and privacy restrictions regarding patient information.

## Abbreviations

The following abbreviations are used in this manuscript:

MDPI	Multidisciplinary Digital Publishing Institute
CVC	Central Venous Catheter
CEA	Cost-Effectiveness Analysis
CMA	Cost-Minimization Analysis
CUA	Cost-Utility Analysis
QALY	Quality-Adjusted Life Years
CBA	Cost-Benefit Analysis
EHR	Electronic Health Records
NNS	number needed to screen
CPI	Consumer Price Index
CT	Computed Tomography
LKF	Leistungsorientierte Krankenanstaltenfinanzierung

## References

[1] Omaghomi T.T., Akomolafe O., Ogugua J.O., Daraojimba A.I., Elufioye O.A. (2024). Healthcare management in a post-pandemic world: Lessons learned and future preparedness-a review. Int. Med. Sci. Res. J..

[2] Porter M. (2006). Redefining Health Care: Creating Value-Based Competition on Results.

[3] Mbau R., Musiega A., Nyawira L., Tsofa B., Mulwa A., Molyneux S., Maina I., Jemutai J., Normand C., Hanson K. (2023). Analysing the efficiency of health systems: A systematic review of the literature. Appl. Health Econ. Health Policy.

[4] Kim D.D., Do L.A., Synnott P.G., Lavelle T.A., Prosser L.A., Wong J.B., Neumann P.J. (2023). Developing criteria for health economic quality evaluation tool. Value Health.

[5] Sailer A.M., van Zwam W.H., Wildberger J.E., Grutters J.P. (2015). Cost-effectiveness modelling in diagnostic imaging: A stepwise approach. Eur. Radiol..

[6] Kjelle E., Brandsæter I.Ø., Andersen E.R., Hofmann B.M. (2024). Cost of low-value imaging worldwide: A systematic review. Appl. Health Econ. Health Policy.

[7] Ziegler K., Feeney J.M., Desai C., Sharpio D., Marshall W.T., Twohig M. (2013). Retrospective review of the use and costs of routine chest x rays in a trauma setting. J. Trauma Manag. Outcomes.

[8] Armao D., Semelka R.C., Elias J. (2012). Radiology’s ethical responsibility for healthcare reform: Tempering the overutilization of medical imaging and trimming down a heavyweight. J. Magn. Reson. Imaging.

[9] Kjelle E., Andersen E.R., Krokeide A.M., Soril L.J., van Bodegom-Vos L., Clement F.M., Hofmann B.M. (2022). Characterizing and quantifying low-value diagnostic imaging internationally: A scoping review. BMC Med. Imaging.

[10] Karel Y.H., Verkerk K., Endenburg S., Metselaar S., Verhagen A.P. (2015). Effect of routine diagnostic imaging for patients with musculoskeletal disorders: A meta-analysis. Eur. J. Intern. Med..

[11] Seong G.M., Lee J., Kim M., Choi J.C., Kim S.W. (2018). Massive air embolism while removing a central venous catheter. Int. J. Crit. Illn. Inj. Sci..

[12] Domínguez D., Reinoso C., Fulla J., Nierga I. (2023). Brain sagging syndrome, a potentially reversible cause of subacute ataxia and dementia. A case report. Neurologia.

[13] Kugiyama T., Koganemaru M., Kuhara A., Nabeta M., Uchiyama Y., Tanaka N., Kawabata M., Abe T. (2018). A rare case of cerebral air embolism caused by pulmonary arteriovenous malformation after removal of a central venous catheter. Kurume Med. J..

[14] Menon G., Jacob R.A., Padmakumar D., George M. (2022). Fragmentation of central venous catheter-A rare but dreaded complication. Indian J. Anaesth..

[15] Dib J., Boutari R., Yaacoub B., Al Khalil N., Al Ayoubi M., Itani O. (2023). Hemothorax Occurring After Central Venous Catheter Removal: A Case Report. Int. J. Clin. Res..

[16] Pinelli F., Muzzi M., Pittiruti M. (2024). Should ultrasound evaluation for catheter-related thrombosis always be required before PICC removal?. J. Vasc. Access.

[17] Nickel B., Gorski L., Kleidon T., Kyes A., DeVries M., Keogh S., Meyer B., Sarver M.J., Crickman R., Ong J. (2024). Infusion therapy standards of practice. J. Infus. Nurs..

[18] Breitwieser M., Moore V., Wiesner T., Wichlas F., Deininger C. (2024). NLP-Driven Analysis of Pneumothorax Incidence Following Central Venous Catheter Procedures: A Data-Driven Re-Evaluation of Routine Imaging in Value-Based Medicine. Diagnostics.

[19] Kjelle E., Andersen E.R., Soril L.J., van Bodegom-Vos L., Hofmann B.M. (2021). Interventions to reduce low-value imaging–a systematic review of interventions and outcomes. BMC Health Serv. Res..

[20] Hendee W.R., Becker G.J., Borgstede J.P., Bosma J., Casarella W.J., Erickson B.A., Maynard C.D., Thrall J.H., Wallner P.E. (2010). Addressing overutilization in medical imaging. Radiology.

[21] Brodersen J. (2017). How to conduct research on overdiagnosis. A keynote paper from the EGPRN May 2016, Tel Aviv: ‘Life can only be understood backwards; but it must be lived forwards’ Søren Kierkegaard (Danish philosopher 1813–1855). Eur. J. Gen. Pract..

[22] Amshel C.E., Palesty J.A., Dudrick S.J., Witherspoon L.E. (1998). Are chest x-rays mandatory following central venous recatheterization over a wire?/Discussion. Am. Surg..

[23] Chui J., Saeed R., Jakobowski L., Wang W., Eldeyasty B., Zhu F., Fochesato L., Lavi R., Bainbridge D. (2018). Is routine chest x-ray after ultrasound-guided central venous catheter insertion choosing wisely?: A population-based retrospective study of 6875 patients. Chest.

[24] Friedmann A.M., Wolfson J.A., Hudson M.M., Weinstein H.J., Link M.P., Billett A., Larsen E.C., Yock T., Donaldson S.S., Marcus K. (2013). Relapse after treatment of pediatric Hodgkin lymphoma: Outcome and role of surveillance after end of therapy. Pediatr. Blood Cancer.

[25] Turchetti G., Kroes M., Lorenzoni V., Trieste L., Chapman A.-M., Sweet A.C., Wilson G.I., Neglia D. (2015). The cost–effectiveness of diagnostic cardiac imaging for stable coronary artery disease. Expert Rev. Pharmacoeconomics Outcomes Res..

[26] Voss S.D., Chen L., Constine L.S., Chauvenet A., Fitzgerald T.J., Kaste S.C., Slovis T., Schwartz C.L. (2012). Surveillance computed tomography imaging and detection of relapse in intermediate-and advanced-stage pediatric Hodgkin’s lymphoma: A report from the Children’s Oncology Group. J. Clin. Oncol..

[27] Neumann P.J., Kim D.D. (2023). Cost-effectiveness thresholds used by study authors, 1990–2021. JAMA.

[28] Vassall A., Sweeney S., Kahn J., Gomez Guillen G., Bollinger L., Marseille E., Herzel B., DeCormier Plosky W., Cunnama L., Sinanovic E. (2017). Reference Case for Estimating the Costs of Global Health Services and Interventions. https://researchonline.lshtm.ac.uk/id/eprint/4653001.

[29] Akram S., Chowdhury Y.S. (2020). Radiation Exposure of Medical Imaging.

[30] Ron E. (2003). Cancer risks from medical radiation. Health Phys..

[31] Baudin C., Vacquier B., Thin G., Chenene L., Guersen J., Partarrieu I., Louet M., Ducou Le Pointe H., Mora S., Verdun-Esquer C. (2023). Occupational exposure to ionizing radiation in medical staff: Trends during the 2009–2019 period in a multicentric study. Eur. Radiol..

[32] McAlister S., McGain F., Breth-Petersen M., Story D., Charlesworth K., Ison G., Barratt A. (2022). The carbon footprint of hospital diagnostic imaging in Australia. Lancet Reg. Health–West. Pac..

[33] Amis E.S., Butler P.F., Applegate K.E., Birnbaum S.B., Brateman L.F., Hevezi J.M., Mettler F.A., Morin R.L., Pentecost M.J., Smith G.G. (2007). American College of Radiology white paper on radiation dose in medicine. J. Am. Coll. Radiol..

[34] Drewett S.R. (2000). Central venous catheter removal: Procedures and rationale. Br. J. Nurs..

[35] Pinelli F., Pittiruti M., Annetta M.G., Barbani F., Bertoglio S., Biasucci D.G., Bolis D., Brescia F., Capozzoli G., D’Arrigo S. (2024). A GAVeCeLT consensus on the indication, insertion, and management of central venous access devices in the critically ill. J. Vasc. Access.

[36] Brindley P., Deschamps J., Milovanovic L., Buchanan B. (2024). Are routine chest radiographs still indicated after central line insertion? A scoping review. J. Intensive Care Soc..

[37] Alrajhi K., Woo M.Y., Vaillancourt C. (2012). Test characteristics of ultrasonography for the detection of pneumothorax: A systematic review and meta-analysis. Chest.

[38] Saugel B., Scheeren T.W., Teboul J.-L. (2017). Ultrasound-guided central venous catheter placement: A structured review and recommendations for clinical practice. Crit. Care.

[39] Smit J.M., Raadsen R., Blans M.J., Petjak M., Van de Ven P.M., Tuinman P.R. (2018). Bedside ultrasound to detect central venous catheter misplacement and associated iatrogenic complications: A systematic review and meta-analysis. Crit. Care.

[40] Ablordeppey E.A., Drewry A.M., Beyer A.B., Theodoro D.L., Fowler S.A., Fuller B.M., Carpenter C.R. (2017). Diagnostic accuracy of central venous catheter confirmation by bedside ultrasound versus chest radiography in critically ill patients: A systematic review and meta-analysis. Crit. Care Med..

